# Striatal dopamine ramping may indicate flexible reinforcement learning with forgetting in the cortico-basal ganglia circuits

**DOI:** 10.3389/fncir.2014.00036

**Published:** 2014-04-09

**Authors:** Kenji Morita, Ayaka Kato

**Affiliations:** ^1^Physical and Health Education, Graduate School of Education, The University of TokyoTokyo, Japan; ^2^Department of Biological Sciences, School of Science, The University of TokyoTokyo, Japan

**Keywords:** dopamine, basal ganglia, corticostriatal, synaptic plasticity, reinforcement learning, reward prediction error, flexibility, computational modeling

## Abstract

It has been suggested that the midbrain dopamine (DA) neurons, receiving inputs from the cortico-basal ganglia (CBG) circuits and the brainstem, compute reward prediction error (RPE), the difference between reward obtained or expected to be obtained and reward that had been expected to be obtained. These reward expectations are suggested to be stored in the CBG synapses and updated according to RPE through synaptic plasticity, which is induced by released DA. These together constitute the “DA=RPE” hypothesis, which describes the mutual interaction between DA and the CBG circuits and serves as the primary working hypothesis in studying reward learning and value-based decision-making. However, recent work has revealed a new type of DA signal that appears not to represent RPE. Specifically, it has been found in a reward-associated maze task that striatal DA concentration primarily shows a gradual increase toward the goal. We explored whether such ramping DA could be explained by extending the “DA=RPE” hypothesis by taking into account biological properties of the CBG circuits. In particular, we examined effects of possible time-dependent decay of DA-dependent plastic changes of synaptic strengths by incorporating decay of learned values into the RPE-based reinforcement learning model and simulating reward learning tasks. We then found that incorporation of such a decay dramatically changes the model's behavior, causing gradual ramping of RPE. Moreover, we further incorporated magnitude-dependence of the rate of decay, which could potentially be in accord with some past observations, and found that near-sigmoidal ramping of RPE, resembling the observed DA ramping, could then occur. Given that synaptic decay can be useful for flexibly reversing and updating the learned reward associations, especially in case the baseline DA is low and encoding of negative RPE by DA is limited, the observed DA ramping would be indicative of the operation of such flexible reward learning.

## Introduction

**Figure 1:**
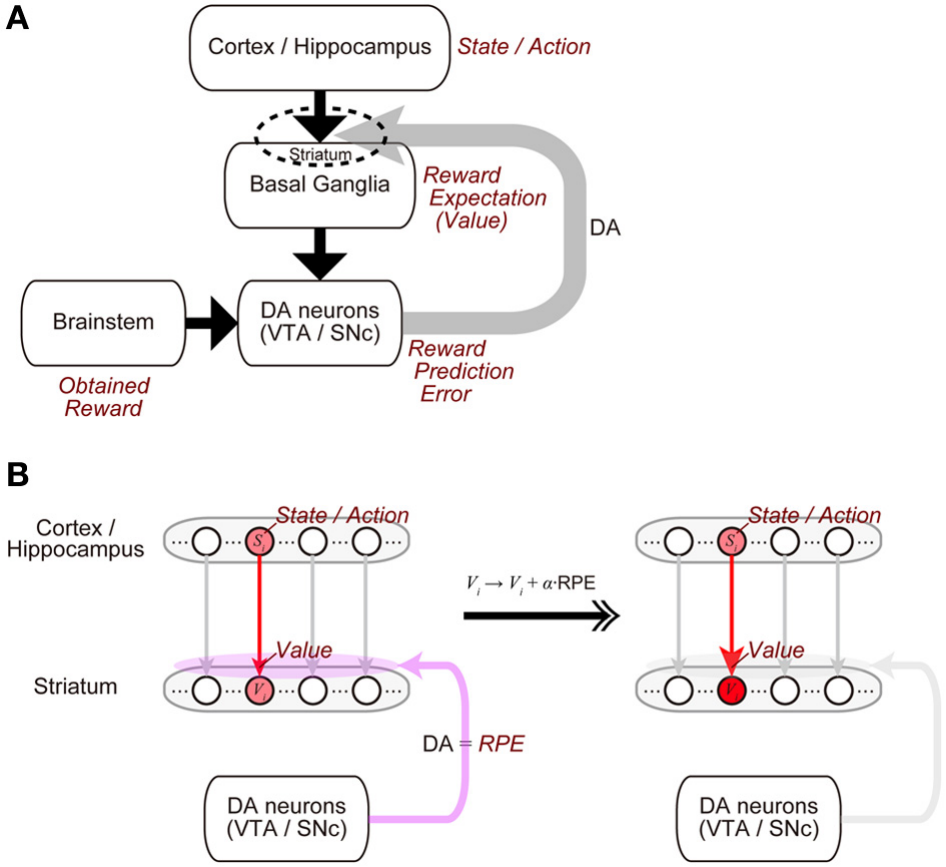
**Mutual interaction between dopamine (DA) and the cortico-basal ganglia (CBG) circuits, and its suggested functional counterpart. (A)** DA neurons in the ventral tegmental area (VTA) and the substantia nigra pars compacta (SNc) receive major inputs from the basal ganglia (BG) and the brainstem. In turn, DA released from these neurons induces plastic changes of synapses in the CBG circuits, in particular, corticostriatal synapses (indicated by the dashed ellipse). This mutual interaction between DA and the CBG circuits has been suggested to implement the algorithm of reinforcement learning as follows. (1) States or actions are represented in the cortex or the hippocampus, and receiving inputs from them, neurons in the BG, in particular, medium spiny neurons in the striatum represent values (reward expectations) of the states/actions, with these values stored in the strengths of the corticostriatal synapses. (2) The DA neurons receive inputs from the BG, as well as inputs from the brainstem, which presumably convey the signal of obtained reward, and compute reward prediction error (RPE). (3) Then, released DA, representing the RPE, induces plastic changes of the corticostriatal synapses, which implement the update of the values (reward expectations) according to the RPE. **(B)** Presumed implementations of processes (1) and (3).

The midbrain dopamine (DA) neurons receive inputs from many brain regions, among which the basal ganglia (BG) are particularly major sources (Watabe-Uchida et al., 2012). In turn, the DA neurons send their axons to a wide range of regions, with again the BG being one of the primary recipients (Björklund and Dunnett, 2007). This anatomical reciprocity between the DA neurons and the BG has been suggested to have a functional counterpart (Figure 1A) (Doya, 2000; Montague et al., 2004; Morita et al., 2013). Specifically, the BG (in particular, the striatum) represents reward expectations, or “values” of stimuli or actions (Kawagoe et al., 2004; Samejima et al., 2005), and presumably influenced by inputs from it, the DA neurons represent the temporal-difference (TD) reward prediction error (RPE), the difference between reward obtained or expected to be obtained and reward that had been expected to be obtained (Montague et al., 1996; Schultz et al., 1997; Steinberg et al., 2013). In turn, released DA induces or significantly modulates plasticity of corticostriatal synapses (Calabresi et al., 1992; Reynolds et al., 2001; Shen et al., 2008) so that the values of stimuli or actions stored in these synapses are updated according to the RPE (Figure 1B). Such a suggested functional reciprocity between the DA neurons and the cortico-BG (CBG) circuits, referred to as the “DA=RPE” hypothesis here, has been guiding research on reward/reinforcement learning and value-based decision-making (Montague et al., 2004; O'Doherty et al., 2007; Rangel et al., 2008; Glimcher, 2011).

Recently, however, Howe et al. (2013) have made an important finding that challenges the universality of the “DA=RPE” hypothesis. Specifically, they have found that, in a reward-associated spatial navigation task, DA concentration in the striatum [in particular, the ventromedial striatum (VMS)] measured by fast-scan cyclic voltammetry (FSCV) primarily shows a gradual increase toward the goal, in both rewarded and unrewarded trials. The “DA=RPE” hypothesis would, in contrast, predict that striatal DA shows a phasic increase at an early timing (beginning of the trial and/or the timing of conditioned stimulus) and also shows a later decrease, rather than an increase, in the case of unrewarded trials (c.f., Niv, 2013).

In most existing theories based on the “DA=RPE” hypothesis, it is assumed that neural circuits in the brain implement mathematical reinforcement learning algorithms in a perfect manner. Behind the request of such perfectness, it is usually assumed, often implicitly, that DA-dependent plastic changes of synaptic strength, which presumably implement the update of reward expectations according to RPE, are quite stable, kept constant without any decay. However, in reality, synapses might be much more dynamically changing, or more specifically, might entail time-dependent decay of plastic changes. Indeed, decay of synaptic potentiation has been observed at least in some experiments examining (presumably) synapses from the hippocampal formation (subiculum) to the ventral striatum (nucleus accumbens) in anesthetized rats (Boeijinga et al., 1993) or those examining synapses in hippocampal slices (Gustafsson et al., 1989; Xiao et al., 1996). Also, active dynamics of structural plasticity of spines has recently been revealed in cultured slices of hippocampus (Matsuzaki et al., 2004). Moreover, functional relevance of the decay of synaptic strength has also been recently put forward (Hardt et al., 2013, 2014). In light of these findings and suggestions, in the present study we explored through computational modeling whether the observed gradual ramping of DA can be explained by extending the “DA=RPE” hypothesis by taking into account such possible decay of plastic changes of the synapses that store learned values. (Please note that we have tried to describe the basic idea of our modeling in the Results so that it can be followed without referring to the Methods.)

## Methods

### Incorporation of decay of learned values into the reinforcement learning model

**Figure 2:**
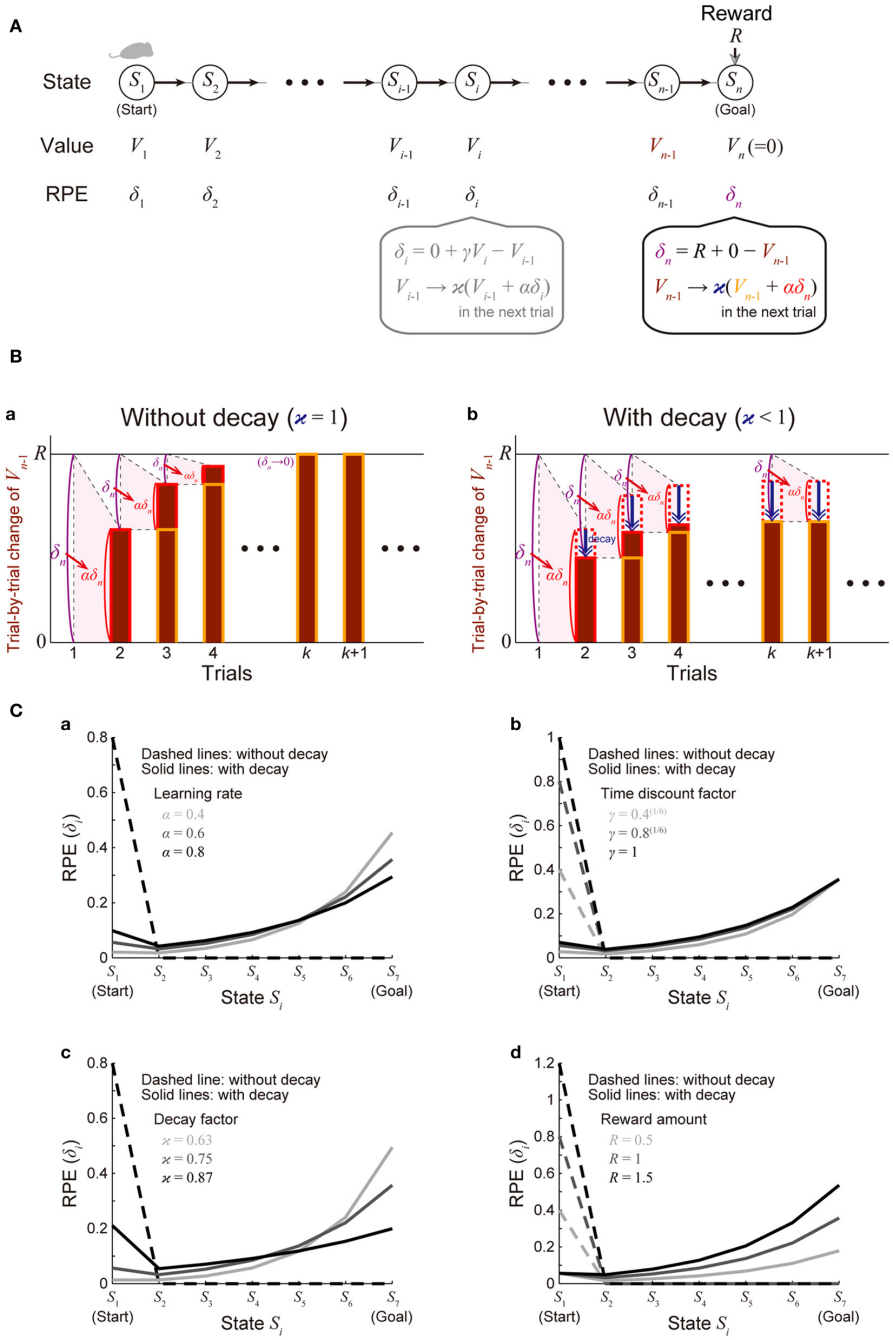
**Incorporation of decay of learned values into the reinforcement learning model causes ramping of RPE. (A)** Simulated spatial navigation (unbranched “I-maze”) task associated with reward. In each trial, subject starts from *S*_1_ (start), and moves to the neighboring state at each time step until reaching *S*_*n*_ (goal), where reward *R*_*n*_ = *R* is obtained. The bottom-middle gray inset shows a pair of computations carried out at each state according to the reinforcement learning model: (I) RPE δ_*i*_ = *R*_*i*_ + γ*V*_*i*_ − *V*_*i* − 1_ is calculated, where *R*_*i*_ is reward obtained at S*i* (*R*_*i*_ = 0 unless *i* = *n*); *V*_*i*_ and *V*_*i* − 1_ are the values of state *S*_*i*_ and *S*_*i* − 1_, respectively; γ (0 ≤ γ ≤ 1) is the time discount factor, and (II) the calculated RPE is used to update the value of *S*_*i* − 1_ : *V*_*i* − 1_ → *ϰ* (*V*_*i* − 1_ + αδ_*i*_), where α (0 ≤ α ≤ 1) is the learning rate and *ϰ* (0 ≤ *ϰ* ≤ 1) is the decay factor: *ϰ* = 1 corresponds to the case of the standard reinforcement model without decay, and *ϰ* < 1 corresponds to the case with decay. The bottom-right inset shows the same computations at the goal (*S*_*n*_): note that *V*_*n*_ is assumed to be 0, indicating that reward is not expected after the goal in a given trial [reward expectation over multiple trials is not considered here for simplicity; it is considered later in the simulations shown in Figure 4 (see the Methods)]. **(B)** Trial-by-trial changes of *V*_*n* − 1_ (value of *S*_*n* − 1_) in the simulated task shown in **(A)**. **(a)** The case of the standard reinforcement learning model without decay [*ϰ* = 1 in **(A)**]. *V*_*n* − 1_ (indicated by the brown bars) gradually increases from trial to trial, and eventually converges to the value of reward (*R*) after many trials while RPE at the goal (δ_*n*_ = *R* + 0 − *V*_*n* − 1_) converges to 0. **(b)** The case of the model incorporating the decay [*ϰ* < 1 in **(A)**]. *V*_*n* − 1_ does not converge to *R* but instead converges to a smaller value, for which the RPE-based increment (αδ_*n*_, indicated by the red dotted/solid rectangles) balances with the decrement due to the decay (indicated by the blue arrows). RPE at the goal (δ_*n*_) thus remains to be positive even after many trials. **(C)** The solid lines show the eventual (asymptotic) values of RPE after the convergence of learning at all the states from the start (*S*_1_) to the goal (*S*_7_) when there are 7 states (*n* = 7) in the model incorporating the decay, with varying **(a)** the learning rate (α), **(b)** the time discount factor (γ), **(c)** the decay factor (*ϰ*), or **(d)** the amount of the reward obtained at the goal (*R*) [unvaried parameters in each panel were set to the middle values (i.e., α = 0.6, γ = 0.8^(1/6)^, *ϰ* = 0.75, and *R* = 1)]. The dashed lines show the cases of the model without decay.

We considered a virtual spatial navigation (unbranched “I-maze”) task as illustrated in Figure 2A. It was assumed that in each trial subject starts from *S*_1_, and moves to the neighboring state in each time step until reaching *S*_*n*_ (goal), where reward *R* is obtained, and subject learns the values of the states through the TD learning algorithm (Sutton and Barto, 1998). For simplicity, first we assumed that there is no reward expectation over multiple trials. Specifically, in the calculation of RPE at *S*_1_ and *S*_*n*_ in every trial, the value of the “preceding state” or the “upcoming state” was assumed to be 0, respectively; later, in the simulations shown in Figure 4, we did consider reward expectation over multiple trials. According to the TD learning, RPE (TD error) at *S*_*i*_ in trial *k* (=1, 2, …), denoted as δ_*i*_ (*k*), is calculated as follows:

$$\delta_{i}\left(k\right)=R_{i}\text{​}\left(k\right)+\gamma V_{i}\text{​}\left(k\right)-V_{i-1}\text{​}\left(k\right),$$

where *V*_*i*_ (*k*) and *V*_*i* − 1_ (*k*) are the value of *S*_*i*_ and state *S*_*i* − 1_ in trial *k*, respectively, *R*_*i*_ (*k*) is the reward obtained at *S*_*i*_ in trial *k* [*R*_*n*_(*k*) = *R* and *R*_*i*_(*k*) = 0 in the other states], and γ (0 ≤ γ ≤ 1) is the time discount factor (per time step). This RPE is used for updating *V*_*i* − 1_(*k*) as follows:

$$V_{i-1}\left(k+1\right)=V_{i-1}\left(k\right)+\alpha \delta_{i}\left(k\right)\text{​},$$

where α (0 ≤ α ≤ 1) represents the learning rate. At the goal (*S*_*n*_) where reward *R* is obtained, these equations are calculated as follows (Figure 2Ba):

$$\begin{array}{l} \;\delta_{n}\text{​}\left(k\right)=R+0-V_{n-1}\left(k\right)\\ V_{n-1}\left(k+1\right)=V_{n-1}\left(k\right)+\alpha \delta_{n}\text{​}\left(k\right)\\ \;=V_{n-1}\left(k\right)+\alpha \text{​}\left\{R-V_{n-1}\left(k\right)\right\}\text{​}, \end{array}$$

given that *V*_*n*_(*k*) = 0 (representing that reward expectation across multiple trials is not considered as mentioned above). In the limit of *k* → ∞ (approximating the situation after many trials) where *V*_*n* − 1_ (*k*) = *V*_*n* − 1_ (*k* + 1) ≡ (denoted as) *V*^∞^_*n* − 1_, the above second equation becomes

$$\begin{array}{l} \;V_{n-1}^{\infty }=V_{n-1}^{\infty }+\alpha \left(R-V_{n-1}^{\infty }\right)\\ ∴V_{n-1}^{\infty }=R \end{array}$$

and therefore

$$\delta_{n}\text{​}\left(k\right)\to R+0-R=0.$$

Similarly, δ_*n* − *j*_ (*j* = 1, 2, 3, …) can be shown to converge to 0 in the limit of *k* → ∞. This indicates that as learning converges, there exists no RPE at any states except for the start (*S*_1_), at which δ_1_(*k*) in the limit of *k* → ∞ is calculated to be γ^*n* − 1^*R*.

Let us now introduce time-dependent decay of the value of the states into the model, in such a way that the update of the state value is described by the following equation (instead of the one described in the above):

$$V_{i-1}\text{​}\left(k+1\right)=\varkappa \text{​}\left\{V_{i-1}\text{​}\left(k\right)+\alpha \delta_{i}\left(k\right)\right\},$$

where *ϰ* (0 < *ϰ* ≤ 1) represents the decay factor (*ϰ* = 1 corresponds to the case without decay). At the goal (*S*_*n*_), this equation is calculated as follows (Figure 2Bb):

$$\begin{array}{l} V_{n-1}\text{​}\left(k+1\right)=\varkappa \left\{V_{n-1}\left(k\right)+\alpha \delta_{n}\left(k\right)\right\}\\ \;=\varkappa V_{n-1}\text{​}\left(k\right)+\alpha \varkappa \left\{R-V_{n-1}\text{​}\left(k\right)\right\}\text{​}. \end{array}$$

In the limit of *k* → ∞ where *V*_*n* − 1_ (*k*) = *V*_*n* − 1_ (*k* + 1) ≡ (denoted as) *V*^∞^_*n* − 1_, this equation becomes

$$\begin{array}{l} V_{n-1}^{\infty }=\varkappa V_{n-1}^{\infty }+\alpha \varkappa \left\{R-V_{n-1}^{\infty }\right\}\\ \;\Leftrightarrow \left\{1-\varkappa \left(1-\alpha \right)\right\}V_{n-1}^{\infty }=\alpha \varkappa R\\ \;\Leftrightarrow V_{n-1}^{\infty }=\alpha \varkappa R/\{1-\varkappa \left(1-\alpha \right)\}, \end{array}$$

and therefore

$$\begin{array}{l} \delta_{n}\left(k\right)\to R+0-\left[\alpha \varkappa R/\{1-\varkappa \left(1-\alpha \right)\}\right]\\ \;=\left[\left(1-\varkappa )/\{1-\varkappa \left(1-\alpha \right)\right\}\right]R\;\left(k\to \infty \right)\text{​}, \end{array}$$

which is positive if *ϰ* is less than 1. This indicates that if there exists decay of the state values, positive RPE remains to exist after learning effectively converges, contrary to the case without decay mentioned above. Similarly, as for the value of *V*_*n* − 2_(*k*) in the limit of *k* → ∞, which we denote *V*^∞^_*n* − 2_,

$$\begin{array}{l} V_{n-2}^{\infty }=\varkappa V_{n-2}^{\infty }+\alpha \varkappa \text{​}\left\{\gamma V_{n-1}^{\infty }-V_{n-2}^{\infty }\right\}\\ \;\Leftrightarrow \{1-\varkappa \text{​}\left(1-\alpha \right)\}V_{n-2}^{\infty }=\alpha \varkappa \gamma V_{n-1}^{\infty }\\ \;=\alpha^{2}\varkappa^{2}\gamma R/\{1-\varkappa \text{​}\left(1-\alpha \right)\}\\ \;\Leftrightarrow V_{n-2}^{\infty }=\alpha^{2}\varkappa^{2}\gamma R/{\left\{1-\varkappa \text{​}\left(1-\alpha \right)\right\}}^{2}\text{​}, \end{array}$$

and therefore

$$\begin{array}{l} \delta_{n-1}\left(k\right)\to 0+\gamma V_{n-1}^{\infty }-V_{n-2}^{\infty }\\ \;=\left[\alpha \varkappa \gamma (1-\varkappa )/{\left\{1-\varkappa \text{​}\left(1-\alpha \right)\right\}}^{2}\right]R\;\left(k\to \infty \right)\text{​}. \end{array}$$

Similarly, in the limit of *k* → ∞, the followings hold for *j* = 1, 2, 3, …, *n* − 2:

$$\begin{array}{l} V_{n-j}\left(k\right)\to V_{n-j}^{\infty }=\alpha^{j}\varkappa^{j}\gamma^{j-1}R/{\left\{1-\varkappa \left(1-\alpha \right)\right\}}^{j},\\ \delta_{n-j}\left(k\right)\to \delta_{n-j}^{\infty }=[\alpha^{j}\varkappa^{j}\gamma^{j}(1-\varkappa )/{\left\{1-\varkappa \left(1-\alpha \right)\right\}}^{j+1}]R. \end{array}$$

At the start of the maze (*S*_1_) (*j* = *n* − 1), the value of the “preceding state” is assumed to be 0 given that reward expectation across multiple trials is not considered as mentioned above, and thus the followings hold in the limit of *k* → ∞:

$$\begin{array}{l} \;V_{n-j}\left(k\right)\to V_{n-j}^{\infty }=\alpha^{j}\varkappa^{j}\gamma^{j-1}R/{\left\{1-\varkappa \left(1-\alpha \right)\right\}}^{j},\\ \delta_{n-j}^{\infty }=0+\gamma V_{n-j}^{\infty }-0=\gamma V_{n-j}^{\infty }\\ \;=\alpha^{j}\varkappa^{j}\gamma^{j}R/{\left\{1-\varkappa \left(1-\alpha \right)\right\}}^{j}. \end{array}$$

The solid lines in Figure 2C show δ^∞^_*i*_ for all the states from the start (*S*_1_) to the goal (*S*_7_) when there are 7 states (*n* = 7), with varying the learning rate (α) (Figure 2Ca), time discount factor (γ) (Figure 2Cb), decay factor (*ϰ*) (Figure 2Cc), or the amount of reward (*R*) (Figure 2Cd) (unvaried parameters in each panel were set to the middle values: α = 0.6, γ = 0.8^(1/6)^, *ϰ* = 0.75, and *R* = 1); the dashed lines show δ^∞^_*i*_ in the model without incorporating the decay for comparison. As shown in the figures, in the cases with decay, the eventual (asymptotic) values of RPE after the convergence of learning entail gradual ramping toward the goal under a wide range of parameters. Also notably, as appeared in the “*ϰ* = 0.87” line in Figure 2Cc, depending on parameters, a peak at the start and a ramp toward the goal could coexist.

### Magnitude-dependent rate of the decay of learned values

We also considered cases where the rate of decay of learned values depends on the current magnitude of values so that larger values are more resistant to decay. We constructed a time-step-based model, in which decay with such magnitude-dependent rate was incorporated. Specifically, we again considered a model of the same I-maze task (Figure 2A) and assumed that RPE is computed at each time step *t* as follows:

δ​(t)=R(t)+γV​(S(t))−V(S(t−1))​,

where *S*(*t*) is the state at time step *t* and *V*(*S*(*t*)) is its value, and *R*(*t*) and *δ*(*t*) are obtained reward and RPE at time step *t*, respectively. γ is the time discount factor (per time step). According to this RPE, the value of state *S*(*t* − 1) was assumed to be updated as follows:

V(S(t−1))→V​(S(t−1))+αδ​(t)​,

where α is the learning rate. We then considered the following function of value *V*:

$$\varkappa \left(V\right)=1-(1-\varkappa_{1})\exp(-V/\varkappa_{2}),$$

where *ϰ*_1_ and *ϰ*_2_ are parameters, and assumed that the value of every state decays at each time step as follows:

*V* → (*ϰ* (*V*))^1/*n*^ × *V* (for the value of states without update according to RPE), or

*V* → (*ϰ* (*V*))^1/*n*^ × (*V* + αδ) (for the value of state with update according to RPE).

**Figure 3:**
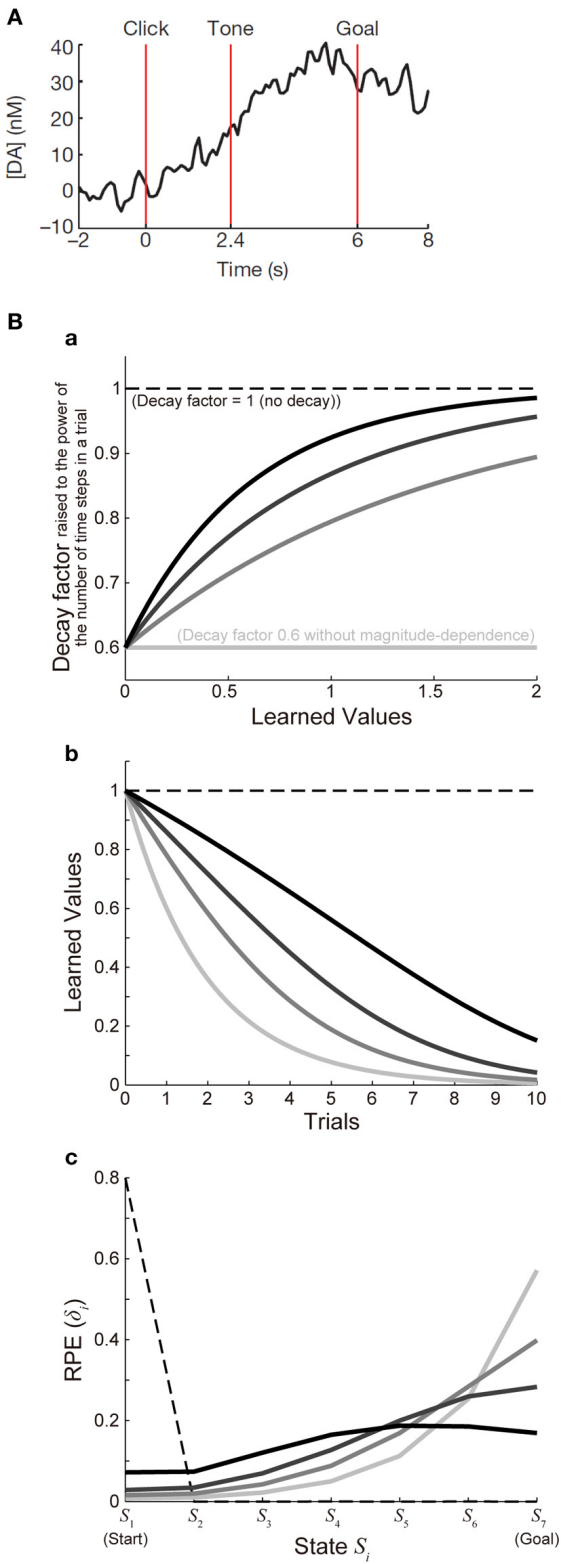
**Decay of learned values with magnitude-dependent rate leads to sigmoidal ramping of RPE resembling the observed DA ramping. (A)** was reprinted by permission from Macmillan Publishers Ltd: Nature (Howe et al., 2013), copyright (2013). **(A)** DA ramping in the ventromedial striatum observed in the experiments (Howe et al., 2013). **(B) (a)** Presumed magnitude-dependence of the rate of decay of learned values raised to the power of the number of time steps in a trial. The horizontal black dashed line at 1 represents the case without decay, and the horizontal lightest-gray solid line at 0.6 represents the case of decay with a constant (magnitude-independent) rate. The three curved lines indicate three different degrees of magnitude-dependence of the rate of decay. **(b)** Decay of learned values under the different degrees of magnitude-dependence of the rate of decay [line colors (brightnesses) correspond to those in panel **(a)**]. **(c)** The solid lines indicate the values of RPE after 100 trials at all the states from the start (*S*_1_) to the goal (*S*_7_) in the simulated I-maze task shown in Figure 2A with 7 states (*n* = 7) in the model incorporating the decay with magnitude-dependent/independent rate, with varying the magnitude-dependence [line colors (brightnesses) correspond to those in **(a,b)**]. The dashed line shows the case of the model without decay.

Figure 3Ba shows the function *ϰ*(*V*) with a fixed value of *ϰ*_1_ (*ϰ*_1_ = 0.6) and various values of *ϰ*_2_ [*ϰ*_2_ = ∞ (lightest gray lines), 1.5 (second-lightest gray lines), 0.9 (dark gray lines), or 0.6 (black lines)], and Figure 3Bb shows the decay of learned values with each of these cases (with 7 time steps per trial assumed). For each of these cases, we simulated 100 trials of the I-maze task shown in Figure 2A with 7 states, with assuming γ = 0.8^(1/6)^ and α = 0.5 and without considering reward expectation over multiple trials, and the eventual values of RPE are presented in the solid lines in Figure 3Bc. Notably, the time-step-based model described in the above is not exactly the same as the trial-based model described in the previous section even for the case where the rate of decay is constant: in the time-step-based model, upon the calculation of RPE: δ(*t*) = *R*(*t*) + γ*V*(*S*(*t*)) − *V*(*S*(*t* − 1)), *V*(*S*(*t*)) has suffered decay (*n* − 1) times, rather than *n* times (which correspond to a whole trial), after it has been updated last time.

### Simulation of maze tasks with rewarded and unrewarded goals

**Figure 4:**
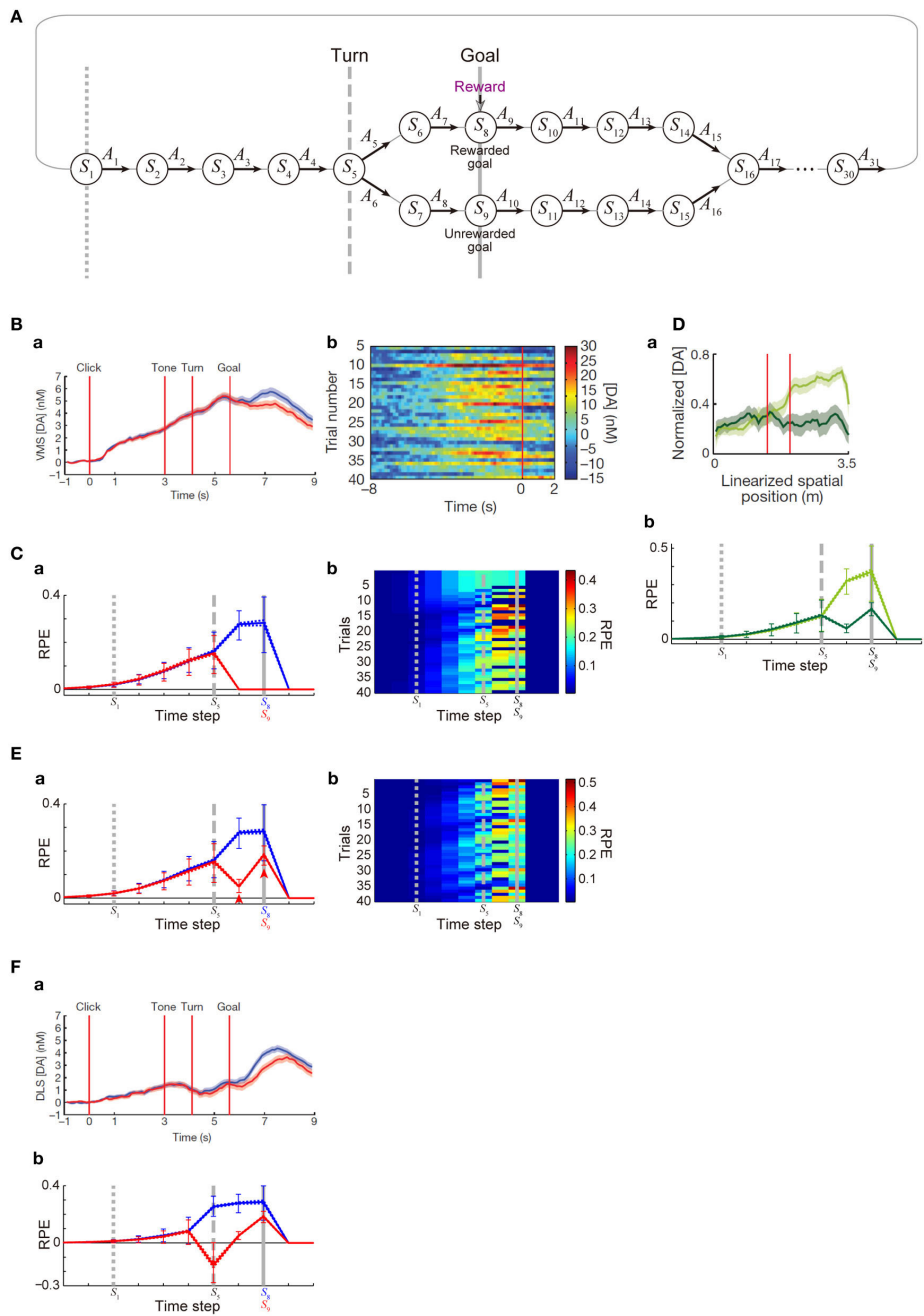
**DA/RPE ramping in maze task with rewarded and unrewarded goals. (Ba,Bb,Da,Fa)** were reprinted by permission from Macmillan Publishers Ltd: Nature (Howe et al., 2013), copyright (2013). **(A)** Simulated free-choice T-maze task with rewarded and unrewarded goals, which was considered as a simplified model of the cue-reward association task used in (Howe et al., 2013). Notably, the boundary between the inter-trial interval and the trial onset was not specifically modeled, and thus there does not exist a particular state that corresponds to the start of each trial. **(B)** Temporal evolution of the DA concentration in the ventromedial striatum in the experiments (Howe et al., 2013). **(a)** Average DA for rewarded (blue) or unrewarded (red) trials. **(b)** Individual trials. **(C)** Temporal evolution of the RPE in the simulations of the model incorporating the decay of learned values with magnitude-dependent rate. **(a)** The thick solid blue and red lines indicate the average, across 25 “pseudo-sessions” (see the Methods), of the mean RPE for rewarded and unrewarded trials in each pseudo-session consisting of 40 trials, respectively. The dotted lines (nearly overlapped with the solid lines) indicate these averages ± s.e.m. across pseudo-sessions. The error bars indicate the average ± standard deviation of RPE in individual trials across trials. The vertical dotted, dashed, and solid gray lines correspond to the lines in **(A)**, indicating *S*_1_, *S*_5_ (branch point), and *S*_8_ or *S*_9_ (goal) in the diagram, respectively. **(b)** Examples of the temporal evolution of RPE in individual trials in the simulations. **(Da)** DA concentration in the forced-choice task in the experiments (Howe et al., 2013). The left red vertical line indicates the branch (choice) point, while the right red line indicates another (unbranched) turning point in the M-maze used in the experiments. **(b)** RPE in the simulations of the simplified forced-choice task by the model. Configurations are the same as those in **(Ca)** except for the colors: light-green and dark-green indicate the large-reward and small-reward cases, respectively. **(E)** RPE in another set of simulations, in which it was assumed that goal-reaching (trial completion) is in itself internally rewarding, specifically, *R*(*t*) in the calculation of RPE (δ(*t*)) at the rewarded goal and the unrewarded goal was assumed to be 1 (external + internal rewards) and 0.25 (internal reward only) [rather than 1 and 0 as in the case of **(C)**], respectively. Configurations are the same as those in **(C)**. **(F) (a)** DA concentration in the dorsolateral striatum in the experiments (Howe et al., 2013). **(b)** RPE in the model incorporating the algorithm called SARSA instead of Q-learning, which was assumed in the simulations shown in **(C,Db,E)**. It was assumed that goal-reaching (trial completion) is in itself internally rewarding in the same manner as in **(E)**. Configurations are the same as those in **(Ca)**.

As a simplified model of the T-maze free-choice task with rewarded and unrewarded goals used in the experiments (Howe et al., 2013) (see the Results for explanation of the task), we considered a free-choice task as illustrated in Figure 4A, where each state represents a relative location on the path expected to lead to, or the path after passing, the rewarded or unrewarded goal or at either of the goals in each trial. We assumed that subject moves to the neighboring state in each time step, and chooses one of the two possible actions (leading to one of the two goals) at the branch point (*S*_5_), while learning the values of each state-action pair (*A*_1_, *A*_2_, ··· : there is assumed to be only a single action “moving forward” in the states other than the branch point), according to one of the major reinforcement (TD) learning algorithms called Q-learning (Watkins, 1989) (for the reason why we have chosen Q-learning, see the Results), with additionally incorporating the decay of learned values with magnitude-dependent rate. Specifically, at each time step *t*, RPE is computed as follows:

$$\begin{array}{l} \delta \text{​}\left(t\right)=R\text{​}\left(t\right)+\gamma Q\left(A\left(t\right)\right)-Q\left(A\left(t-1\right)\right)\left(\text{at states other than }S_{5}\right)\\ \delta \text{​}\left(t\right)=R\text{​}\left(t\right)+\gamma \text{max}\left\{Q\left(A_{5}\right),Q\left(A_{6}\right)\right\}-Q\left(A\left(t-1\right)\right)\left(\text{at }S_{5}\right)\text{​}, \end{array}$$

where *A*(*t*) is the state-action pair at time step *t* and *Q*(*A*(*t*)) is its value, and γ is the time discount factor (per time step). There were assumed to be *N* = 25 time steps per trial, including the inter-trial interval, and γ was set to γ = 0.8^1/25^. According to this RPE, the value of the previous state-action pair is updated as follows:

Q(A(t−1))→Q(A(t−1))+αδ(t)​,

where α is the learning rate and it was set to 0.5. We then assumed that the value of every state-action pair (denoted as *Q*) decays at each time step as follows:

$$Q\to {\left(\varkappa \left(Q\right)\right)}^{1/N}\times Q,$$

where *ϰ* (*Q*) is the function introduced above, and *ϰ*_1_ and *ϰ*_2_ were set to *ϰ*_1_ = 0.6 and *ϰ*_2_ = 0.6. At the branch point (*S*_5_), one of the two possible actions (*A*_5_ and *A*_6_) is chosen according to the following probability:

$$\begin{array}{l} \text{Prob}\left(A_{5}\right)=1/\left(1+\exp\left(-\beta \left(Q\left(A_{5}\right)-Q\left(A_{6}\right)\right)\right)\right)\text{​},\\ \text{Prob}\left(A_{6}\right)=1/\left(1+\exp\left(-\beta \left(Q\left(A_{6}\right)-Q\left(A_{5}\right)\right)\right)\right)\\ \;=1-\text{Prob}\left(A_{5}\right)\text{​}, \end{array}$$

where Prob(*A*_5_) is the probability that action *A*_5_ is chosen, and β is a parameter determining the degree of exploration vs. exploitation upon choice (as β becomes smaller, choice becomes more and more exploratory); β was set to 1.5. In the simulations of this model, we considered reward expectation over multiple trials, specifically, we assumed that at the first time step in every trial, subject moves from the last state in the previous trial to the first state in the current trial, and RPE computation and value update are done in the same manner as in the other time steps.

In addition to the simulations of the Q-learning model, we also conducted simulations of the model with a different algorithm called SARSA (Rummery and Niranjan, 1994) (the results shown in Figure 4F), for which we assumed the following equation for the computation of RPE at the branch point (*S*_5_):

$$\delta \text{​}\left(t\right)=R\text{​}\left(t\right)+\gamma Q\left(A_{\text{chosen}}\right)-Q\left(A\left(t-\text{1}\right)\right)\text{​},$$

where *A*_chosen_ is the action that is actually chosen (either *A*_5_ or *A*_6_), instead of the equation for Q-learning described above. In the simulations shown in Figure 4C, reward *R*(*t*) was assumed to be 1 only at one of the goals (*S*_8_) and set to 0 otherwise, whereas in the simulations shown in Figure 4E and Figure 4F, R(*t*) was assumed to be 1 and 0.25 at the two goals (*S*_8_ and *S*_9_, respectively) and set to 0 otherwise. In addition to the modeling and simulations of the free-choice task, we also conducted simulations of a forced-choice task, which could be regarded as a simplified model of the forced-choice task examined in the experiments (Howe et al., 2013). For that, we considered sequential movements and action selection in the same state space (Figure 4A) but randomly determined choice (*A*_5_ or *A*_6_) at the branch point (*S*_5_) in each trial rather than using the choice probability function described above (while RPE of the Q-learning type, taking the max of *Q*(*A*_5_) and *Q*(*A*_6_), was still assumed), and reward *R*(*t*) at the two goals were set to 1 (large reward) and 0.25 (small reward). In each of the conditions, 1000 trials were simulated, with initial values of *Q*(*A*) set to 0 for every state-action pair *A*. We did not specifically model sessions, but we considered that the 1000 trials were divided into 25 “pseudo-sessions,” each of which consists of 40 trials, so as to calculate the average and s.e.m. of the mean RPE in individual pseudo-sessions across the 25 pseudo-sessions, which are shown in the solid and dashed lines in Figures 4Ca,Db,Ea,Fb (in these figures, the average ± standard deviation of RPE in individual trials across trials are also shown in the error bars). Figures 4Cb,Eb show the RPE in 401st ~ 440th trials. In the simulations of 1000 trials for Figures 4C,D,E by the Q-learning model with decay, negative RPE did not occur. By contrast, negative RPE occurred rather frequently in the SARSA model (Figure 4F). The ratio that the rewarded goal (*S*_8_) was chosen (i.e., ratio of correct trials) was 65.6, 64.5, and 64.5% in the simulations of 1000 trials for Figures 4C,E,F, respectively. The simulations in the present work were conducted by using MATLAB (MathWorks Inc.), and the program codes will be submitted to the ModelDB (https://senselab.med.yale.edu/modeldb/).

## Results

### Decay of plastic changes of synapses leads to ramping of RPE-representing DA signal

We will first show how the standard reinforcement learning algorithm called the TD learning (Sutton and Barto, 1998) works and what pattern of RPE is generated by using a virtual reward learning task, and thereafter we will consider effects of possible decay of plastic changes of synapses storing learned values. We considered a virtual spatial navigation task as illustrated in Figure 2A. In each trial, subject starts from *S*_1_, and moves to the neighboring state in each time step until reaching the goal (*S*_*n*_), where reward *R* is obtained (unbranched “I-maze,” rather than branched “T-maze,” was considered first for simplicity). Based on the prevailing theories of neural circuit mechanisms for reinforcement learning (Montague et al., 1996; Doya, 2000), we have made the following assumptions: (1) different spatial locations, or “states,” denoted as *S*_1_ (=start), *S*_2_, ···, *S*_*n*_ (=goal, where reward *R* is obtained), are represented by different subpopulations of neurons in the subject's brain (hippocampus and/or cortical regions connecting with it), and (2) “values” of these states are stored in the changes (from the baseline) in the strength of synapses between the state-representing neurons in the cortex/hippocampus and neurons in the striatum (c.f. Pennartz et al., 2011), and thereby the value of a given state *S*, denoted as *V*(*S*), is represented by the activity of a corresponding subpopulation of striatal neurons. We have further assumed, again based on the current theories, that the following pair of computations are carried out at each state (*S*_*i*_, *i* = 1, 2, …, *n*) in the DA-CBG system: (I) DA neurons receive (indirect) impacts from the striatal neurons through basal ganglia circuits, and compute the TD RPE: δ_*i*_ = *R*_*i*_ + γ*V*_*i*_ − *V*_*i* − 1_, where *R*_*i*_ is reward obtained at S*i* (*R*_*i*_ = 0 unless *i* = *n*); *V*_*i*_ and *V*_*i* − 1_ are the “values” (meaning reward expectations after leaving the states) of state *S*_*i*_ and *S*_*i* − 1_, respectively; and γ (0 ≤ γ ≤ 1) is a parameter defining the degree of temporal discount of future rewards called the time discount factor, and (II) the RPE is used to update the value of the previous state (i.e., *S*_*i* − 1_) through DA-dependent plastic changes of striatal synapses: *V*_*i* − 1_ → *ϰ* (*V*_*i* − 1_ + αδ_*i*_), where α (0 ≤ α ≤ 1) represents the speed of learning called the learning rate, and *ϰ* (0 ≤ *ϰ* ≤ 1) is a parameter for the time-dependent decay; we first considered the case of the standard reinforcement learning model without decay (the case with *ϰ* = 1).

Assume that initially subject does not expect to obtain reward after completion of the maze run in individual trials and thus the “values” of all the states are 0. When reward is then introduced into the task and subject obtains reward *R*_*n*_ = *R* at the goal (*S*_*n*_), positive RPE δ_*n*_ = *R* + γ*V*_*n*_ − *V*_*n* − 1_ = *R* + 0 − 0 = *R* occurs, and it is used to update the value of *S*_*n* − 1_ : *V*_*n* − 1_ → 0 + αδ_*n*_ = α*R*. Then, in the next trial, subject again obtains reward *R* at the goal (S_*n*_) and positive RPE occurs; this time, the RPE amounts to δ_*n*_ = *R* + γ*V*_*n*_ − *V*_*n* − 1_ = *R* + 0 − α*R* = (1 − α)*R*, and it is used to update the value of *S*_*n* − 1_ : *V*_*n* − 1_ → α*R* + αδ_*n*_ = (2 α − α^2^) *R*. In this way, the value of *S*_*n* − 1_ (*V*_*n* − 1_) gradually increases from trial to trial, and accordingly RPE occurred at the goal (δ_*n*_ = *R* − *V*_*n* − 1_) gradually decreases. As long as *V*_*n* − 1_ is smaller than *R*, positive RPE should occur and *V*_*n* − 1_ should increase in the next trial, and eventually, *V*_*n* − 1_ converges to *R*, and RPE (δ_*n*_) converges to 0 (Figure 2Ba) (see the Methods for mathematical details). Similarly, values of the preceding states except for the initial state (V_*n* − 1_, *V*_*n* − 2_, ···; except for *V*_1_) also converge to *R* and RPE at these states (δ_*n* − 1_, δ_*n* − 2_, ···; except for δ_1_) converges to 0. Thus, from the prevailing theories of neural circuit mechanisms for reinforcement learning, it is predicted that DA neuronal response at the timing of reward and the preceding timings except for the initial timing, representing the RPE δ_*n*_, δ_*n* − 1_, δ_*n* − 2_, ···, appears only transiently when reward is introduced into the task (or the amount of reward is changed), and after that transient period DA response appears only at the initial timing, as shown in the dashed lines in Figure 2C, which indicate eventual (asymptotic) values of RPE in the case with 7 states, with various parameters. The gradual ramping of DA signal observed in the actual reward-associated spatial navigation task (Howe et al., 2013) therefore cannot be explained by the DA=RPE hypothesis standing on the standard reinforcement (TD) learning algorithm (Niv, 2013).

Let us now assume that DA-dependent plastic changes of synaptic strengths are subject to time-dependent decay so that learned values stored in them decay with time. Let us consider a situation where *V*_*n* − 1_ (value of *S*_*n* − 1_) is smaller than *R* and thus positive RPE occurs at *S*_*n*_. If there is no decay, *V*_*n* − 1_ should be incremented exactly by the amount of this RPE multiplied by the learning rate (α) in the next trial, as seen above (Figure 2Ba). If there is decay, however, *V*_*n* − 1_ should be incremented by the amount of α × RPE but simultaneously decremented by the amount of decay. By definition, RPE (δ_*n*_ = *R* − *V*_*n* − 1_) decreases as *V*_*n* − 1_ increases. Therefore, if the rate (or amount) of decay is constant, *V*_*n* − 1_ could initially increase from its initial value 0 given that the net change of *V*_*n* − 1_ per trial (i.e., α × RPE − decay) is positive, but then the net change per trial becomes smaller and smaller as *V*_*n* − 1_ increases, and eventually, as α × RPE becomes asymptotically equal to the amount of decay, increase of *V*_*n* − 1_ should asymptotically terminate (Figure 2Bb). Even at this asymptotic limit (approximating the situation after many trials), RPE at the goal (δ_*n*_) remains to be positive, because it should be equal to the amount of decay divided by α. Similarly, RPE at the timings preceding reward (δ_*n* − 1_, δ_*n* − 2_, ···) also remains to be positive (see the Methods for mathematical details). The situation is thus quite different from the case without decay, in which RPE at the goal and the preceding timings except for the initial timing converges to 0 as seen above. The solid lines in Figure 2C show the eventual (asymptotic) values of RPE in the I-maze task (Figure 2A) with 7 states in the case of the model with decay, amount of which is assumed to be proportional to the current magnitude of the state value (synaptic strength) (i.e., the *rate* of decay is constant, not depending on the magnitude), with varying the learning rate (α) (Figure 2Ca), the time discount factor (γ) (Figure 2Cb), the decay factor (κ) (Figure 2Cc), or the amount of reward (*R*) (Figure 2Cd). As shown in the figures, under a wide range of parameters, RPE entails gradual ramping toward the goal, and the ramping pattern is proportionally scaled with the amount of reward (Figure 2Cd).

### Explanation of the observed gradually ramping DA signal

As shown so far, the experimentally observed gradual ramping of DA concentration toward the goal could potentially be explained by incorporating the decay of plastic changes of synapses storing learned values into the prevailing hypothesis that the DA-CBG system implements the reinforcement learning algorithm and DA represents RPE. In the following, we will see whether and how detailed characteristics of the observed DA ramping can be explained by this account. First, the experimentally observed ramping of DA concentration in the VMS entails a nearly sigmoidal shape (Figure 3A) (Howe et al., 2013), whereas the pattern of RPE/DA ramping predicted from the above model (Figure 2C) is just convex downward, with the last part (just before the goal) being the steepest. We explored whether this discrepancy can be resolved by elaborating a model. In the model considered in the above, we assumed decay with a constant (magnitude-independent) rate. In reality, however, the rate of decay may depend on the magnitude of learned values (synaptic strengths storing the values). Indeed, it has been shown in hippocampal slices that longer tetanus trains cause a larger degree of long-term potentiation, which tends to exhibit less decay (Gustafsson et al., 1989). Also, in the experiments examining (presumably) direct inputs from the hippocampal formation (subiculum) to the nucleus accumbens (Figure 6A of Boeijinga et al., 1993), decay of potentiation appears to be initially slow and then accelerated. We constructed an elaborate model incorporating decay with magnitude-dependent rate, which could potentially be in accord with these findings. Specifically, in the new model we assumed that larger values (stronger synapses) are more resistant to decay (see the Methods for details). We simulated the I-maze task (Figure 2A) with this model, and examined the eventual values of RPE after 100 trials, with systematically varying the magnitude-dependence of the rate of decay (Figures 3Ba,b). Figure 3Bc shows the results. As shown in the figure, as the magnitude-dependence of the rate of decay increases so that larger values (stronger synapses) become more and more resistant to decay, the pattern of RPE ramping changes its shape from purely convex downward to nearly sigmoidal. Therefore, the experimentally observed nearly sigmoidal DA ramping could be better explained by tuning such magnitude-dependence of the rate of decay.

Next, we examined whether the patterns of DA signal observed in the free-choice task (Howe et al., 2013), specifically, cue (tone)—reward association T-maze task, can be reproduced by our model incorporating the decay. In that task, subject started from the end of the trunk of letter “T”. As the subject moved forward, a cue tone was presented. There were two different cues (1 or 8 kHz) indicating which of the two goals lead to reward in the trial. Subject was free to choose either the rewarded goal or the unrewarded goal. In the results of the experiments, subjects chose the rewarded (“correct”) goal in more than a half (65%) of trials overall, indicating that they learned the cue-reward association and made advantageous choices at least to a certain extent. During the task, DA concentration in the VMS was shown to gradually ramp up, in both trials in which the rewarded goal was chosen and those in which the unrewarded goal was chosen, with higher DA concentration at late timings observed in the rewarded trials (Figure 4Ba). We tried to model this task by a simplified free-choice task as illustrated in Figure 4A, where each state represents a relative location on the path expected to lead to, or the path after passing, the rewarded or unrewarded goal or at either of the goals in each trial. The VMS, or more generally the ventral striatum receives major dopaminergic inputs from the DA neurons in the ventral tegmental area (VTA), whose activity pattern has been suggested (Roesch et al., 2007) to represent a particular form of RPE defined in one of the major reinforcement (TD) learning algorithms called Q-learning (Watkins, 1989). Therefore, we simulated sequential movements and action selection in the task shown in Figure 4A by using the Q-learning model incorporating the decay of learned values with magnitude-dependent rate (see the Methods for details).

Given that the model's parameters are appropriately tuned, the model's choice performance can become comparable to the experimental results (about 65% correct), and the temporal evolution of the RPE averaged across rewarded trials and also the average across unrewarded trials can entail gradual ramping during the trial (Figure 4Ca), reproducing a prominent feature of the experimentally observed DA signal. In the experiments (Howe et al., 2013), the authors have shown that the moment-to-moment level of DA during the trial is likely to reflect the proximity to goal (location in the maze) rather than elapsed time. Although our model does not have description of absolute time and space, the value of RPE in our model is uniquely determined depending on the state, which is assumed to represent relative location in the maze, and thus given that the duration of DA's representation of RPE co-varies with the duration spent in each state, our model could potentially be consistent with the observed insensitivity to elapsed time. A major deviation of the simulated RPE/DA from the experimentally observed DA signal is that difference between rewarded trials and unrewarded trials is much larger in the simulation results, as appeared in Figure 4Ba and Figure 4Ca. We will explore how this could be addressed below. Figure 4Cb shows examples of the temporal evolution of RPE in individual trials in the simulations. As appeared in the figure, ramping can occur in a single trial at least for a certain fraction of trials, although more various patterns, including ramping peaked at earlier times, transient patterns, and patterns with more than one peaks, also frequently appear (see Figure 4Bb for comparison with the experimental results). Closely looking at the simulation results (Figure 4Cb), there exist oblique stripe patterns from top right to bottom left (especially clearly seen for blue colors), indicating that upward or downward deviation of RPE values, first occurred at the timing of goal and the preceding timing due to presence or absence of reward, transmits to earlier timing (to the left in the figure) in subsequent trials (to the bottom). The reason for the appearance of such a pattern is that RPE is used to update the value of state-action pair at the previous timing. This pattern is a prediction from the model and is expected to be experimentally tested, although the difference in DA signal around the timing of goal between rewarded and unrewarded trials was much smaller in the experiments, as mentioned above, and thus finding such a pattern, even if exist, would not be easy.

In the study that we modeled (Howe et al., 2013), in addition to the free-choice task, the authors also examined a forced-choice task, in which subject was pseudo-randomly forced to choose one of the goals associated with high or low reward in each trial. The authors have then found that DA ramping was strongly biased toward the goal with the larger reward (Figure 4Da). We considered a simplified model of the forced-choice task, represented as state transitions in the diagram shown in Figure 4A with the two goals associated with large and small rewards and the choice in each trial determined (pseudo-)randomly (see the Methods for details). We conducted simulations of this task by using our model with the same parameters used in the simulations of the free-choice task, and found that the model could reproduce the bias toward the goal with the larger reward (Figure 4Db).

### Explanation of further features of the observed DA signal

Although our model could explain the basic features of the experimentally observed DA ramping to a certain extent, there is also a major drawback as mentioned in the above. Specifically, in our simulations of the free-choice task, gradual ramping of the mean RPE was observed in both the average across rewarded trials and the average across unrewarded trials, but there was a prominent difference between these two (Figure 4Ca). In particular, whereas the mean RPE for rewarded trials ramps up until subject reaches the goal, the mean RPE for unrewarded trials ramps up partway but then drops to 0 after passing the branch point. In the experiments (Howe et al., 2013), the mean RPE for rewarded trials and that for unrewarded trials did indeed differentiate later in a trial (Figure 4Ba), but the difference was much smaller, and the timing of differentiation was much later, than the simulation results. The discrepancy in the timing could be partially understood given that our model describes the temporal evolution of RPE, which is presumably first represented by the activity (firing rate) of DA neurons whereas the experiments measured the concentration of DA presumably released from these neurons and thus there is expected to be a time lag, as suggested from the observed difference in latencies of DA neuronal firings (Schultz et al., 1997) and DA concentration changes (Hart et al., 2014). The discrepancy in the size of the difference between rewarded and unrewarded trials, however, seems not to be explained in such a straightforward manner even partially. In the following, we would like to present a possible explanation for it.

In the simulations shown in the above, it was assumed that the unrewarded goal is literally not rewarding at all. Specifically, in our model, we assumed a positive term representing obtained reward (*R*(*t*) > 0) in the calculation of RPE (δ(*t*)) at the rewarded goal, but not at the unrewarded goal [where *R*(*t*) was set to 0]. In reality, however, it would be possible that reaching a goal (completion of a trial) is in itself internally rewarding for subjects, even if it is the unrewarded goal and no external reward is provided. In order to examine whether incorporation of the existence of such internal reward could improve the model's drawback that the difference between rewarded and unrewarded trials is too large, we conducted a new simulation in which a positive term representing obtained external or internal reward (*R*(*t*) > 0) was included in the calculation of RPE (δ(*t*)) at both the rewarded goal and the unrewarded goal, with its size four times larger in the rewarded goal [i.e., *R(t)* = 1 or 0.25 at the rewarded or unrewarded goal, respectively; this could be interpreted that external reward of 0.75 and internal reward of 0.25 are obtained at the rewarded goal whereas only internal reward of 0.25 is obtained at the unrewarded goal]. Figure 4E shows the results. As shown in Figure 4Ea, the mean RPE averaged across unrewarded trials now remains to be positive after the branch point and ramps up again toward the goal (arrowheads in the figure), and thereby the difference between rewarded and unrewarded trials has become smaller than the case without internal reward. Neural substrate of the presumed positive term (*R*(*t*)) representing internal reward is not sure, but given the suggested hierarchical reinforcement learning in the CBG circuits (Ito and Doya, 2011), such inputs might originate from a certain region in the CBG circuits that controls task execution and goal setting (in the outside of the part that is modeled in the present work).

In the study that we modeled (Howe et al., 2013), DA concentration was measured in both the VMS and the dorsolateral striatum (DLS), and there was a difference between them. In the VMS, nearly constant-rate ramping starts just after the trial-onset, and rewarded and unrewarded trials differentiate only in the last period, as we have seen above (Figure 4Ba). In the DLS, by contrast, initial ramping looks less prominent than in the VMS, while rewarded and unrewarded trials appear to differentiate somewhat earlier than in the VMS (Figure 4Fa). The VMS and DLS, or more generally the ventral striatum and dorsal striatum, are suggested to receive major dopaminergic inputs from the VTA and the substantia nigra pars compacta (SNc), respectively (Ungerstedt, 1971), though things should be more complicated in reality (Björklund and Dunnett, 2007; Bromberg-Martin et al., 2010). Both VTA and SNc DA neurons have been shown to represent RPE, but they may represent different forms of RPE used for different reinforcement (TD) learning algorithms. Specifically, it has been empirically suggested, albeit in different species, that VTA and SNc DA neurons represent RPE for Q-learning (Roesch et al., 2007) and SARSA (Morris et al., 2006), respectively; these two algorithms differ in whether the maximum value of all the choice options (Q-learning) or the value of actually chosen option (SARSA) is used for the calculation of RPE [see (Niv et al., 2006) and the Methods]. Conforming to this suggested distinction, so far we have assumed Q-learning in the model and compared the simulation results with the DA concentration in the VMS that receives major inputs from the VTA. The emerging question, then, is whether simulation results become more comparable to the DA concentration in the DLS if we instead assume SARSA in the model. We explored this possibility by conducting a new simulation, and found that it would indeed be the case. Figure 4Fb shows the simulation results of the model with SARSA, which also incorporated the internal reward upon reaching the unrewarded goal introduced above. Compared with the results with Q-leaning (Figure 4Ea), initial ramping looks less prominent, and rewarded and unrewarded trials differentiate earlier. These two differences could be said to be in line with the experimentally observed differences between the VMS and DLS DA concentrations as described above, although again the difference between rewarded and unrewarded trials is larger, and the timing of differentiation is earlier, in the model than in the experiment.

Intriguingly, in the study that has shown the representation of RPE for Q-learning in VTA DA neurons (Roesch et al., 2007), DA neurons increased their activity in a staggered manner from the beginning of a trial (before cue presentation) toward reward, with the activity in the middle of the increase shown to entail the characteristics of RPE. It is tempting to guess that such a staggered increase of VTA DA neuronal firing actually has the same mechanistic origin as the gradual increase of VMS DA concentration in the study that we modeled (Howe et al., 2013). Consistent with this possibility, in a recent study that has simulated the experiments in which VTA DA neurons were recorded (Roesch et al., 2007) by using a neural circuit model of the DA-CBG system (Morita et al., 2013), the authors have incorporated decay of learned values, in a similar manner to the present work, in order to reproduce the observed temporal pattern of DA neuronal firing, in particular, the within-trial increase toward reward (although it was not the main focus of that study and also the present work does not rely on the specific circuit structure/mechanism for RPE computation proposed in that study).

## Discussion

While the hypothesis that DA represents RPE and DA-dependent synaptic plasticity implements update of reward expectations according to RPE has become widely appreciated, recent work has revealed the existence of gradually ramping DA signal that appears not to represent RPE. We explored whether such DA ramping can be explained by extending the “DA=RPE” hypothesis by taking into account possible time-dependent decay of DA-dependent plastic changes of synapses storing learned values. Through simulations of reward learning tasks by the RPE-based reinforcement learning model, we have shown that incorporation of the decay of learned values can indeed cause gradual ramping of RPE and could thus potentially explain the observed DA ramping. In the following, we discuss limitations of the present work, comparisons and relations with other studies, and functional implications.

### Limitations of the present work

In the study that has found the ramping DA signal (Howe et al., 2013), it was shown that the peak of the ramping signals was nearly as large as the peak of transient responses to unpredicted reward. By contrast, in our simulations shown in Figure 4E, average RPE for all the trials at state *S*_5_ is about 0.158, which is smaller than RPE for unpredicted reward of the same size in our model (it is 1.0). This appears to deviate from the results of the experiments. However, there are at least three potential reasons that could explain the discrepancy between the experiments and our modeling results, as we describe below.

First, in the experiments, whereas there was only a small difference between the peak of DA response to free reward and the peak of DA ramping during the maze task when averaged across sessions, the slope of the regression line between these two values (DA ramping / DA to free reward) in individual sessions (Extended Data Figure 5a of Howe et al., 2013) is much smaller than 1 (it is about 0.26). Indeed, that figure shows that there were rather many sessions in which the peak of DA response to free reward was fairly large (>15 nM) whereas the peak of DA ramping during the maze task was not large (<15 nM), while much less sessions exhibited the opposite pattern. How the large variability in DA responses in the experiments reflects heterogeneity of DA cells and/or other factors is not sure, but it might be possible to regard our model as a model of cells or conditions in which response to free reward was fairly large whereas ramping during the maze task was not large. Second, it is described in Howe et al. (2013) (legend of Extended Data Figure 5a) that DA response to free reward was compared with DA ramping measured from the same probes during *preceding* behavioral training in the maze. Given that the same type of reward (chocolate milk) was used in the task and as free reward, and that the measurements of DA response to deliveries of free reward were made after the measurements of DA ramping during 40 maze-task trials in individual sessions, we would think that there possibly existed effects of satiety. Third, the degree of unpredictability of the “unexpected reward” in the experiments could matter. Specifically, it seems possible that there were some sensory stimuli that immediately preceded reward delivery and informed the subjects of it such as sounds (generated in the device for reward supply) or smells. In such a case, conventional RPE models without decay predict that, after some experience of free reward, RPE of nearly the same size as that of RPE generated upon receiving ultimately unpredictable reward is generated at the timing of the sensory stimuli (unless time discount is extremely severe: size becomes smaller only due to time discount), and no RPE is generated at the timing of actual reward delivery. In contrast, and crucially, our model with decay predicts that, after some experience of free reward, RPE generated at the timing of the sensory stimuli is significantly smaller than RPE generated upon receiving ultimately unpredictable reward, and positive RPE also occurs upon receiving reward but it is also smaller than the ultimately unpredictable case (if the timing of the sensory stimuli is one time-step before the timing of reward in the model with the parameters used for Figure 4, RPE values at those two timings after 15 experiences are about 0.87 and 0.16, respectively; these two RPEs are about 0.99 and 0.00 in the case without decay). The mechanism of this can be schematically understood from Figure 2Bb by viewing *V*_*n* − 1_ (bar height) and δ_*n*_ (space above the bar) as RPEs at the timings of the preceding sensory stimuli and the actual reward delivery, respectively (as for the former, except for time discount); they are both smaller than the reward amount (“*R*”), which is the size of RPE generated upon receiving this reward ultimately unpredictably. With these considerations, we would think that the discrepancy between the experiments and the model in the relative sizes of the peak DA response to free reward and the peak DA ramping in the maze task could potentially be explained.

Other than the point described above, there are at least six fundamental limitations of our model. First, our model's behavior is sensitive to the magnitude of rewards. As shown in the Results, in our original model assuming decay with a constant rate, overall temporal evolution of RPE is proportionally scaled according to the amount of reward (Figure 2Cd). However, such a scalability no longer holds for the elaborated model incorporating the magnitude-dependent rate of decay, because the assumed magnitude-dependence (Figure 3Ba) is sensitive to absolute reward amount. Consequently, the patterns of RPE shown in Figures 3 and 4 will change if absolute magnitude of rewards is changed. In reality, it is possible that magnitude-dependence of the rate of decay of learned values (synaptic strength) itself can be changed, in a longer time scale, depending on the average magnitude of rewards obtained in the current context. Second, whereas the free-choice task used in the experiments (Howe et al., 2013) involved cue-reward association, our simplified model does not describe it. Because of this, the state in our model is assumed to represent relative location on the path expected to lead to, or the path after passing, the rewarded or unrewarded goal or at either of the goals in each trial (as described before), but not absolute location since the absolute location of rewarded/unrewarded goal in the experiments was determined by the cue, which changed from trial to trial. Third, our model only has abstract representation of relative time and space, and how they are linked with absolute time and space is not defined. Fourth, validity of our key assumption that plastic changes of synapses are subject to time-dependent decay remains to be proven. There have been several empirical suggestions for the (rise and) decay of synaptic potentiation (Gustafsson et al., 1989; Boeijinga et al., 1993; Xiao et al., 1996) and spine enlargement (Matsuzaki et al., 2004) in the time scale of minutes, which could potentially fit the time scale of the maze task simulated in the present study, but we are currently unaware of any reported evidence for (or against) the occurrence of decay of DA-dependent plastic changes of synapses in animals engaged in tasks like the one simulated in the present study. Also, we assumed simple equations for the decay, but they would need to be revised in future works. For example, any plastic changes will eventually decay back to 0 according to the models in the present work, but in reality at least some portion of the changes is likely to persist for a long term as shown in the experiments referred to in the above. Fifth, regarding the origin of the ramping DA signal and its potential relationships with the DA=RPE hypothesis, there are potentially many possibilities, and the mechanism based on the decay of learned values proposed in the present study is no more than one of them (see the next section for two of other possibilities). Sixth, potential modulation of DA release apart from DA neuronal firing is not considered in the present study. We have assumed that the observed ramping DA signal in the striatum (Howe et al., 2013) faithfully reflects DA neuronal firing, which has been suggested to represent RPE. However, as pointed out previously (Howe et al., 2013; Niv, 2013), whether it indeed holds or not is yet to be determined, because DA neuronal activity was not measured in that study and DA concentration can be affected by presynaptic modulations of DA release, including the one through activation of nicotinic receptors on DA neuronal axons by cholinergic interneurons (Threlfell et al., 2012), and/or saturation of DA reuptake. Addressing these limitations would be interesting topics for future research.

### Comparisons and relations with other studies

Regarding potential relationships between the ramping DA signal in the spatial navigation task and the DA=RPE hypothesis, a recent theoretical study (Gershman, 2014) has shown that DA ramping can be explained in terms of RPE given nonlinear representation of space. This is an interesting possibility, and it is entirely different from our present proposal. The author has argued that his model is consistent with important features of the observed DA ramping, including the dependence on the amount of reward and the insensitivity to time until the goal is reached. Both of these features could also potentially be consistent with our model, although there are issues regarding the sensitivity of model's behavior to reward magnitude and the lack of representation of absolute time and space, as we have so far described. It remains to be seen whether the limitations of our model, including the large difference between rewarded and unrewarded trials, are not the case with his model. Notably, these two models are not mutually exclusive, and it is possible that the observed DA ramping is a product of multiple factors. Also, the possible correspondence between the differential DA signal in the ventral vs. dorsal striatum and Q-learning vs. SARSA mentioned in the Results could also hold with Gershman's model.

It has also been shown (Niv et al., 2005) that the conventional reinforcement learning model (without decay) can potentially explain ramping of averaged DA neuronal activity observed in a task with probabilistic rewards (Fiorillo et al., 2003), if it is assumed that positive and negative RPEs are asymmetrically represented by increase and decrease of DA neuronal activity from the baseline, with the dynamic range of the decrease narrower than that of the increase due to the lowness of the baseline firing rate. This mechanism did not contribute to the ramping of RPE in our simulations, because such asymmetrical representation was not incorporated into our model; actually, negative RPE did not occur in the 1000-trials simulations of our Q-learning model for Figures 4C,Db,E, while negative RPE occurred rather frequently in the SARSA model (Figure 4Fb). Notably, according to the mechanism based on the asymmetrical RPE representation by DA (Niv et al., 2005), ramping would not appear in the I-maze task where reward is obtained in every trial without uncertainty (Figure 2A) because negative RPE would not occur in such a situation, different from the cases of the decay-based mechanism proposed in the present work and the mechanism proposed by Gershman (Gershman, 2014) mentioned above. Experimental examination of the I-maze would thus be potentially useful to distinguish mechanisms that actually operate. In the meantime, the mechanism based on the asymmetrical RPE representation by DA is not mutually exclusive with the other two, and two or three mechanisms might simultaneously operate in reality.

### Functional implications

Given that the observed DA ramping is indicative of decay of learned values as we have proposed, what is the functional advantage of such decay? Decay would naturally lead to forgetting, which is rather disadvantageous in many cases. However, forgetting can instead be useful in certain situations, in particular, where environments are dynamically changing and subjects should continually overwrite old memories with new ones. Indeed, it has recently been proposed that decay of plastic changes of synapses might be used for active forgetting (Hardt et al., 2013, 2014). Inspired by this, here we propose a possible functional advantage of synaptic decay specifically for the DA-CBG system involved in value learning. In value learning, active forgetting is required when associations between rewards and preceding sensory stimuli are changed, such as the case of reversal learning in which cue-reward association is reversed unpredictably. In theory, flexible reversal of leaned association should be possible based solely on RPE without any decay: old association can be erased by negative RPE first, and new association can then be learned by positive RPE. However, in reality there would be a problem due to a biological constraint. Specifically, it has been indicated that the dynamic range of DA neuronal activity toward the negative direction from the baseline firing rate is much narrower than the positive side, presumably for the sake of minimizing energy cost (c.f., Laughlin, 2001; Bolam and Pissadaki, 2012; Pissadaki and Bolam, 2013), and thereby DA neurons can well represent positive RPE, but perhaps not negative RPE (Bayer and Glimcher, 2005) (see also Potjans et al., 2011). This indication has been challenged by subsequent studies: it has been shown (Bayer et al., 2007) that negative RPE was correlated with the duration of pause of DA neuronal firing, and a recent study using FSCV (Hart et al., 2014) has shown that DA concentration in the striatum in fact symmetrically encoded positive and negative RPE in the range tested in that study. Nevertheless, it could still be possible that representation of negative RPE by DA is limited in case the baseline DA concentration is low. In such a case, synaptic decay could be an alternative or additional mechanism for erasing old, already irrelevant cue-reward associations so as to enable flexible reversal/reconstruction of associations, with possibly the rate of decay itself changing appropriately (i.e., speeding up just after the reversal/changes in the environments) through certain mechanisms (e.g., monitoring of the rate of reward acquisition). We thus propose that decay of learned values stored in the DA-dependent plastic changes of CBG (corticostriatal) synapses would be a feature of the DA-CBG circuits, which endows the reinforcement learning system with flexibility, in a way that is also compatible with the minimization of energy cost.

With such consideration, it is suggestive that DA ramping was observed in the study using the spatial navigation task (Howe et al., 2013) but not in many other studies (though there could be symptoms as we discussed above). Presumably, it reflects that the spatial navigation task is ecologically more relevant, for rats, than many other laboratory tasks. In the wild, rats navigate to forage in dynamically changing environments, where flexibility of learning would be pivotal. Moreover, the overall rate of rewards in wild foraging would be lower than in many laboratory tasks, and given the suggestion that the rate of rewards is represented by the background concentration of DA (termed tonic DA) (Niv et al., 2007), tonic DA in foraging rats is expected to be low and thus representation of negative RPE by DA could be limited as discussed above. The rate of decay of learned values would therefore be adaptively set to be high so as to turn on the alternative mechanism for flexible learning, and it would manifest as the prominent ramping of DA/RPE in the task mimicking foraging navigation (even if the rate of rewards is not that low in the task, different from real foraging). If this conjecture is true, changing the volatility of the task, mimicking changes in the volatility of the environment, may induce adaptive changes in the rate of decay of learned values (synaptic strengths), which could cause changes in the property of DA ramping (c.f., Figure 2Cc): a testable prediction of our model.

Apart from the decay, DA ramping can also have more direct functional meanings. Along with its roles in plasticity induction, DA also has significant modulatory effects on the responsiveness of recipient neurons. In particular, DA is known to modulate the activity of the two types of striatal projection neurons to the opposite directions (Gerfen and Surmeier, 2011). Then, given that DA neurons compute RPE based on value-representing BG inputs, on which the activity of striatal neurons have direct and/or indirect impacts, ramping DA, presumably representing a gradual increase of RPE according to our model, would modulate the activity of striatal neurons and thereby eventually affect the computation of RPE itself. Such a closed-loop effects (c.f., Figure 1A) can potentially cause rich nonlinear phenomena through recurrent iterations. Exactly what happens depends on the precise mechanism of RPE computation, while the present work does not assume specific mechanism for it so that the results presented so far can generally hold. Just as an example, however, when the model of the present study is developed into a model of the DA-CBG circuit based on a recently proposed mechanism for RPE computation (Morita et al., 2012, 2013; Morita, 2014), consideration of the effects of DA on the responsiveness of striatal projection neurons can lead to an increase in the ratio of correct trials, indicating occurrence of positive feedback (unpublished observation). This could potentially represent self-enhancement of internal value or motivation (c.f., Niv et al., 2007). Such an exciting possibility is also expected to be explored in future work.

## Author contributions

Kenji Morita conceived and designed the research. Kenji Morita and Ayaka Kato performed the modeling, calculations, and simulations. Kenji Morita drafted the manuscript. Ayaka Kato commented on the manuscript, and contributed to its revision and elaboration.

### Conflict of interest statement

The authors declare that the research was conducted in the absence of any commercial or financial relationships that could be construed as a potential conflict of interest.
