# Corrigendum: Striatal dopamine ramping may indicate flexible reinforcement learning with forgetting in the cortico-basal ganglia circuits

**DOI:** 10.3389/fncir.2014.00048

**Published:** 2014-05-16

**Authors:** Kenji Morita, Ayaka Kato

**Affiliations:** ^1^Physical and Health Education, Graduate School of Education, The University of TokyoTokyo, Japan; ^2^Department of Biological Sciences, School of Science, The University of TokyoTokyo, Japan

**Keywords:** dopamine, basal ganglia, corticostriatal, synaptic plasticity, reinforcement learning, reward prediction error, flexibility, computational modeling

In the preparation of organized program codes for this article (Morita and Kato, 2014) for submission to public database after the publication, we have noticed that there was an error in the code for making Figure 2Cd written by one of the authors Kenji Morita. Specifically, although RPE values at *S*_1_ for the cases with decay (i.e., the leftmost points of the three solid lines) should be proportional to the amount of reward as appeared in the formula for calculating them:

$$\begin{array}{l} \left(\text{at the start of the maze }(S_{1}\right)\\ \text{(j = n - 1))}\\ \delta_{n-j}^{\;\infty }=0+\gamma V_{n-j}^{\;\infty }-0\\ \;=\gamma V_{n\;-j}^{\;\infty }\\ \;=\alpha^{j}\varkappa^{j}\gamma^{j}R/{\left\{1-\varkappa (1-\alpha )\right\}}^{j}\\ \;(\text{in the right-bottom of page 4}), \end{array}$$

where “*R*” represents the amount of reward, they were incorrectly plotted as an equal value in Figure 2Cd (indicated by the red circle in the left (“Error”) panel of the figure attached to this Corrigendum) because “*R*” was mistakenly dropped (i.e., effectively assumed to be 1 in all the cases) in the code. We have corrected the code and made the corrected Figure 2Cd [the right (“Corrected”) panel of the figure attached to this Corrigendum]. There is no need to change the texts explaining Figure 2Cd in the Methods, Results, and the figure legend. We sincerely apologize for the inconvenience. Lastly, we would like to take this opportunity to announce that the program (MATLAB) codes for this article (with the correction described in the above) are now available on the ModelDB (Accession: 153573): http://senselab.med.yale.edu/modeldb/ShowModel.asp?model=153573

**Figure 2:**
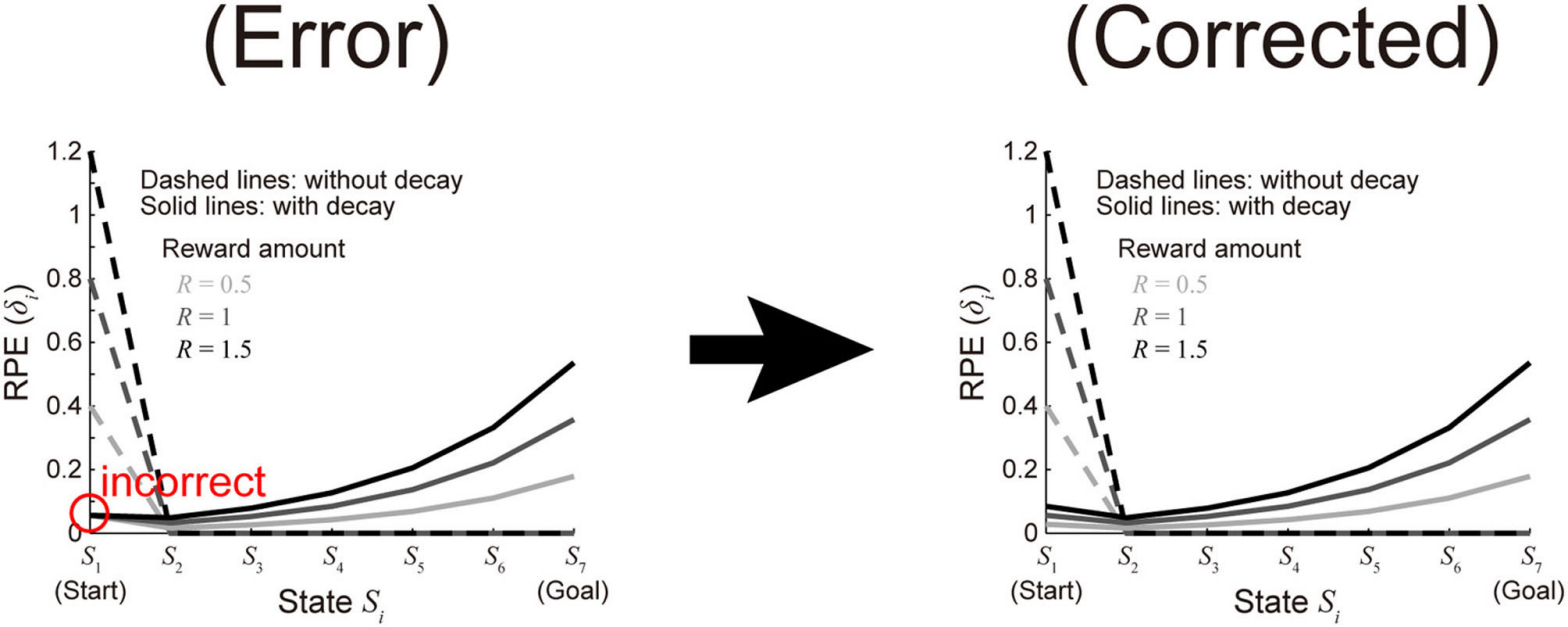
**(C) (d)** The solid lines show the eventual (asymptotic) values of RPE after the convergence of learning at all the states from the start (*S*_1_) to the goal (*S*_7_) when there are 7 states (*n* = 7) in the model incorporating the decay, with varying the amount of the reward obtained at the goal (*R*) (unvaried parameters were set as follows: *a* = 0.6, *y* = 0.8^(1/6)^, and *x* = 0.75). The dashed lines show the cases of the model without decay.

## Conflict of interest statement

The authors declare that the research was conducted in the absence of any commercial or financial relationships that could be construed as a potential conflict of interest.
